# Effectiveness and Safety of Warfarin in Dialysis Patients With Atrial Fibrillation: A Meta-Analysis of Observational Studies

**DOI:** 10.1097/MD.0000000000002233

**Published:** 2015-12-18

**Authors:** Gang Liu, Ming Long, Xun Hu, Cheng-Heng Hu, Xin-Xue Liao, Zhi-Min Du, Yu-Gang Dong

**Affiliations:** From the Department of Cardiology, the First Affiliated Hospital, Sun Yat-Sen University, Guangzhou, China.

## Abstract

In routine practice, warfarin is widely used in dialysis patients with atrial fibrillation (AF) for stroke prevention though the ratio of risks to benefits remains unclear. Recent cohort studies investigating the association between warfarin use and the risks of stroke and bleeding in dialysis patients with AF present conflicting results.

The objective of this study was to assess the effectiveness and safety of warfarin use in patients with AF undergoing dialysis.

Three databases PubMed, EMBASE, and OVID were searched from their inception to August 2015.

Observational studies which assessed the ischemic stroke or bleeding risk of warfarin use in dialysis patients with AF were included. Two reviewers independently extracted data and assessed methodological quality based on the Newcastle–Ottawa Scale score. Combined hazard ratios (HRs) and 95% conﬁdence intervals (CIs) were calculated using the random-effects model and heterogeneity was assessed based on the Cochrane Q-statistic test and the *I*^2^ statistic. Metaregression analyses were performed to explore the source of heterogeneity.

A total of 11 eligible studies with 25,407 patients were included in the analysis. Warfarin use, in comparison with no-warfarin use, was not associated with a lower risk for ischemic stroke (HR 0.95, 95% CI 0.66–1.35). Sensitivity analyses found results to be robust. Metaregression analysis showed that demographic feature, clinical characteristics, or study-level variable had no impact of warfarin use on stroke risk. In addition, warfarin use was associated with a 27% higher risk for bleeding (95% CI 1.04–1.54). Overall, warfarin use did not have a significant association with reduced mortality (95% CI 0.96–1.11).

It appears that warfarin use is not beneficial in reducing stroke risk, but with a high risk for bleeding in dialysis patients with AF. Randomized trials are needed to determine the risk-benefit ratio of warfarin in dialysis patients with AF.

## INTRODUCTION

Atrial fibrillation (AF) is an increasingly common comorbidity in patients with end-stage renal disease (ESRD), which requires treatment with dialysis.^1^ Epidemiologic studies showed that 7% to 20% of dialysis patients have recurrent or permanent AF.^1,2^ The activation of inflammatory pathways and elevation of oxidative stress may play important roles in the pathogenesis of AF in dialysis patients.^3,4^ Dialysis patients with AF experience a 5-fold higher risk for a new stroke.^5^ As warfarin has been proved to effectively reduce the risk of stroke in the general population,^6,7^ it is often prescribed in such dialysis patients for stroke prevention.^8^ However, the risk-benefit ratio of warfarin therapy in dialysis patients should be adequately assessed because warfarin has been shown to accelerate vascular calcification in patients with ESRD, which potentially induce an increased stroke risk.^9,10^ Current epidemiologic literature regarding warfarin use and the risk for ischemic stroke and bleeding in patients with AF undergoing dialysis presents conflicting results.^11–21^ Therefore, we systematically examined the effectiveness and safety of warfarin use in dialysis patients with AF. To our knowledge, there was no published randomized trial specifically related to this topic.

## METHODS

### Ethics Statement

As this study is a meta-analysis, ethical approval was not required.

### Data Sources

According to the meta-analysis of Observational Studies in Epidemiology guidelines,^22^ we systematically searched PubMed, EMBASE, and OVID database using the following keywords: “warfarin,” “atrial fibrillation,” “chronic renal (kidney) insufficiencies (failure),” “chronic kidney disease,” “end-stage renal disease,” “dialysis,” “renal (kidney) dialysis,” or “hemodialysis.” The search was limited to human research with no restrictions on language. Reference lists of all identified studies and review articles were hand-searched for relevant citations. The final search was run on August 30, 2015.

### Study Selection

Studies were included if they met the following criteria: (1) prospective or retrospective studies assessing the stroke or bleeding risk of warfarin use in dialysis patients with AF; (2) describe the definition for stroke and bleeding; (3) described the match or adjustment for confounding variables; and (4) reported relative risk or hazard ratio (HR) estimates with confidence intervals (CIs). Conference abstracts were included if detailed data on stroke or bleeding risk were reported.

### Data Extraction

All data extraction was performed independently by 2 reviewers (GL, ML). The following information was obtained from each study: (1) first author's last name, year of publication, and country of origin; (2) study design and sample size; (3) definition for stroke and bleeding; (4) effect size estimates with 95% CIs, and the variables for match or adjustment.

### Quality Assessment

The quality of each study was assessed independently by 2 reviewers (GL, ML) using the Newcastle–Ottawa Scale (NOS)^23^ Any discrepancies were resolved by consensus. The NOS was made up of 3 different dimensions—selection, comparability, and outcomes or exposure. The NOS assigns a maximum of 4 points for selection, 2 for comparability, and 3 for outcomes or exposure. A score above 6 was considered as high quality.

### Data Analysis

Our meta-analysis and statistical analyses were performed by STATA 12.0 (STATA Corp. LP, College Station, TX). A *P* value < 0.05 was considered statistically signiﬁcant, unless otherwise specified. The summary HRs from individual studies calculated using the generic inverse variance method and using the random-effects model of DerSimonian-Laird. We chose the random-effects method because of its conservative summary estimate and because it incorporates between- and within-study variance. Measures of association were combined under the assumption that HRs were accurate approximations of relative risks. Heterogeneity was measured using the Cochrane Q-statistic test and the *I*^2^ statistic: for the Q statistic, a *P* value <0.1 was considered statistically signiﬁcant for heterogeneity, whereas for *I*^2^, a value >50% was considered significant heterogeneity.^24^ Publication bias was assessed visually by funnel plots, and statistically by a regression asymmetry test (Egger test).

Potential sources of heterogeneity were explored using univariate metaregression analyses and considered the following study-level demographic and clinical variables: age, sex, follow-up, study design, sample size, year of publication, coronary artery disease, congestive heart failure, diabetes mellitus, hypertension, and prior stroke. In addition, the sensitivity analysis was performed to test the robustness of the results.

## RESULTS

### Study Selection

**FIGURE 1:**
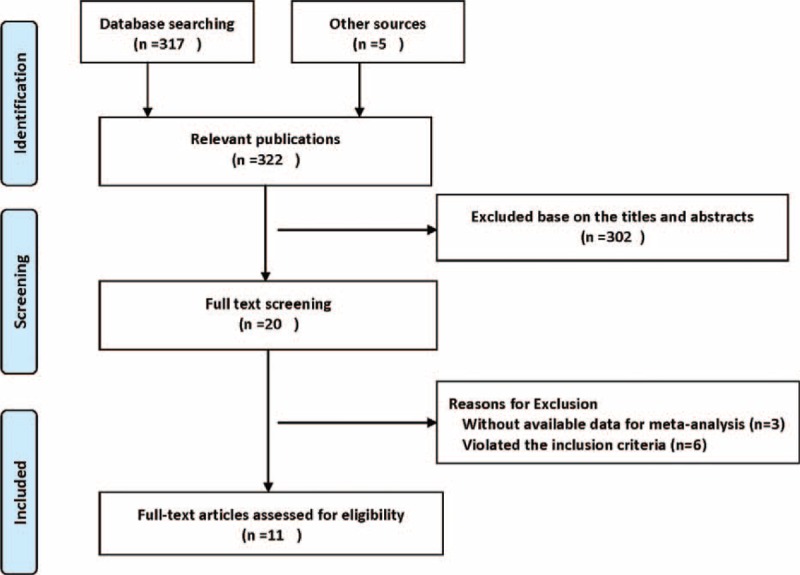
Search strategy and flowchart for studies included in the meta-analysis.

A total of 322 records were identified during the initial search. After screening the titles and abstracts of these citations, 20 full-text articles were retrieved for detailed evaluation. Nine literatures were eliminated for the reasons that 6 of them violated the inclusion criteria and 3 without available data for the meta-analyses. Detailed selection process was showed in Figure 1. It is noteworthy that no clinical randomized trial was found to specifically assess the risk–benefit ratio of warfarin in dialysis patients with AF.

### Study Characteristics

Eleven eligible cohort studies were identified, consisting of 2 prospective^12,15^ and 9 retrospective studies^11,13,14,16–21^ and involving a total of 25,407 patients. Participants were followed-up for 1.6 to 15 years and the studies have been published between 2003 and 2015. The main characteristics of the selected studies and the definition of stroke and bleeding are presented in Table 1. The patient characteristics of included studies are summarized in Table 2. Mean age for participants was 70.2 years. Overall, the comorbidities, such as coronary artery disease, congestive heart failure, diabetes mellitus, and hypertension, were common among these patients. In addition, these patients had a high risk score for stroke (CHADS2 or CHADS2-VASc score≥2).

**TABLE 1:**
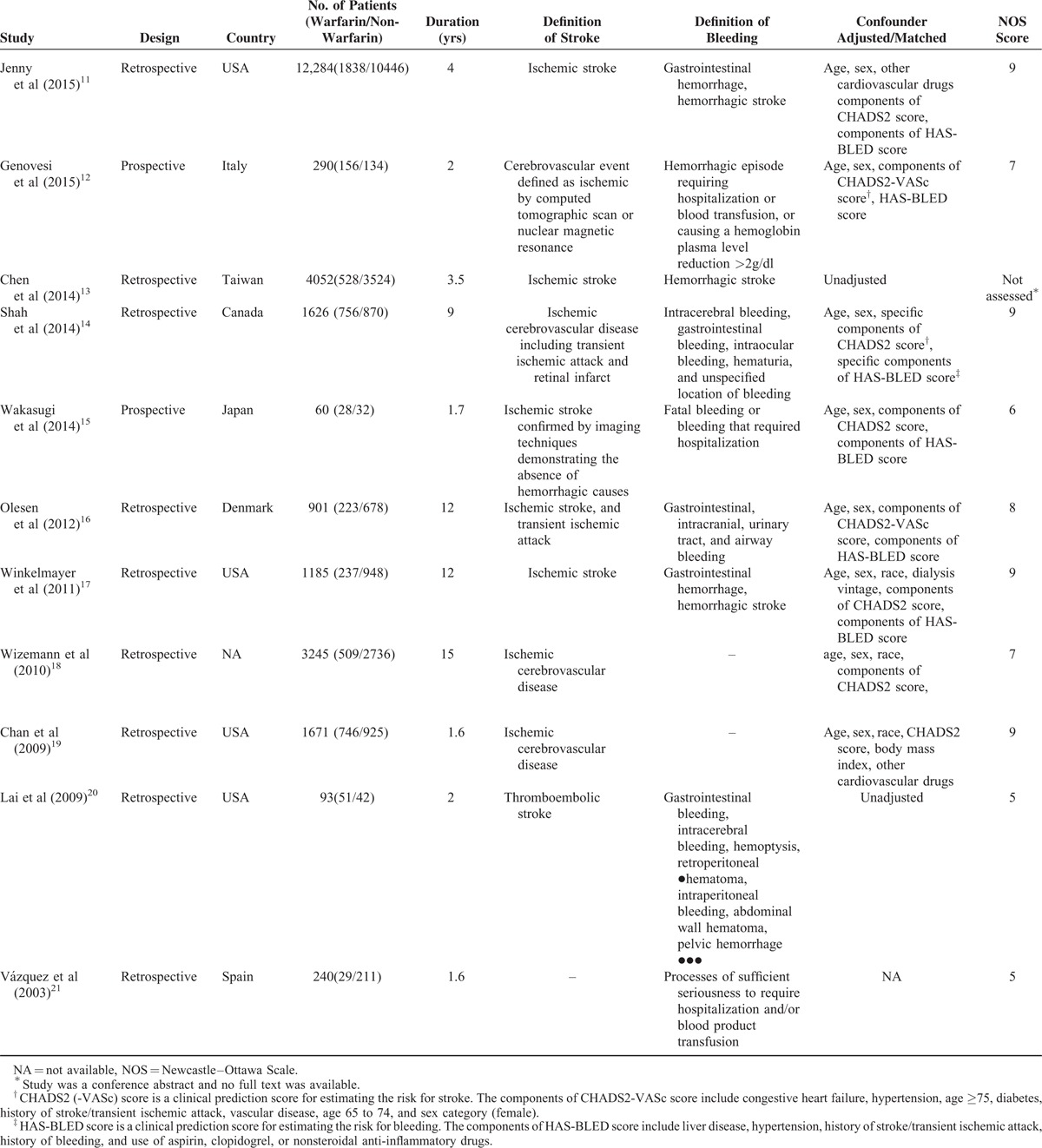
Characteristics of Included Studies

**TABLE 2:**
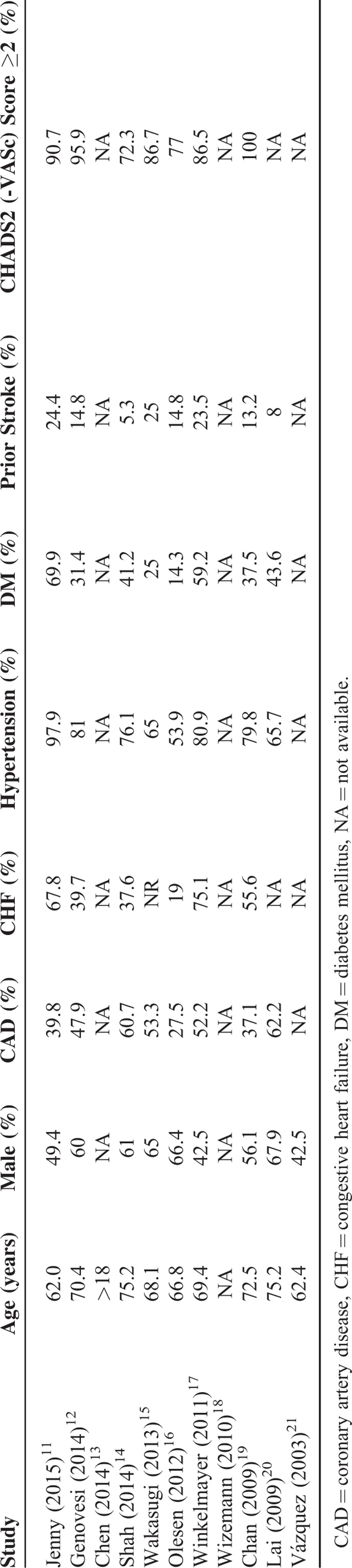
The Patient Characteristics of Included Studies

### Quality of Included Studies

Our assessment of study quality revealed that small observational studies were prone to a high risk of biases. Representativeness of included patients was limited in 4 studies,^12,15,20,21^ which may lead to indication bias. Of the 4 studies, 2 adjusted the baseline imbalance in important confounders and the remaining 2 did not.^20,21^ In addition, the presence of non-negligible loss to follow-up in 3 studies^15,16,18^ may lead to survivor bias and distort the result. Overall, based on the NOS, 8 studies^11,12,14–19^ were of high quality, and 2^20,21^ of low quality.

### Association of Warfarin Use and Risk of Ischemic Stroke

**FIGURE 2:**
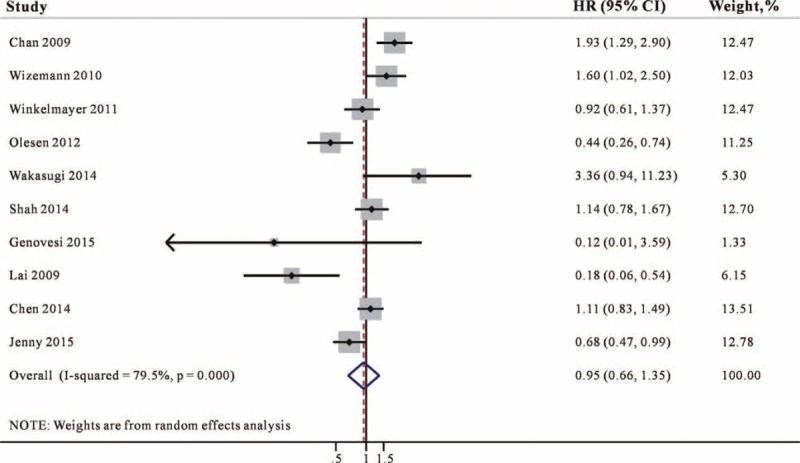
Combined estimate of hazard ratios (HRs) and 95% confidence intervals (CIs) of warfarin use associated with stroke risk. Squares represent the HRs (size of square reflects the study's weight), and lines represent the 95% CIs for individual studies. The diamond represents the pooled HR and 95% CI. CI = confidence intervals, HRs =  hazard ratios.

Ten studies^11–20^ evaluated the associations between warfarin use and risk of stroke in AF patients undergoing dialysis, of which 8^11,12,14–19^ used the multivariable Cox proportional hazards models to estimate the HR of incident stroke, which controlled for the major confounders (namely age, sex, and specific components of CHADS2 or CHADS2-VASc score), whereas the remaining 2^13,20^ only reported unadjusted RR. In the meta-analysis of risk for stroke, because of significant heterogeneity (*P* < 0.001, *I*^2^ = 76.4%), which was to be expected due to some studies showing positive, no, or negative association, a random-effects model was chosen over a fixed-effects model. A pooled analysis of 10 studies found that warfarin use, in comparison with no-warfarin use, was not associated with a lower risk for ischemic stroke in dialysis patients (HR 0.95, 95% CI 0.66–1.35, Figure 2). Furthermore, the risk of stroke was not significantly altered even after adjusting for potential confounders where reported in studies (pooled adjusted HR [95% CI] = 1.05 [0.70–1.58]). Meta-regression analysis showed that no demographic feature, clinical characteristic, or study-level variable modified the impact of warfarin use on stroke risk. In sensitivity analyses, the HRs were similar without great fluctuation, confirming the stability of the present result (data not shown).

### Association of Warfarin Use and Bleeding Risk

**FIGURE 3:**
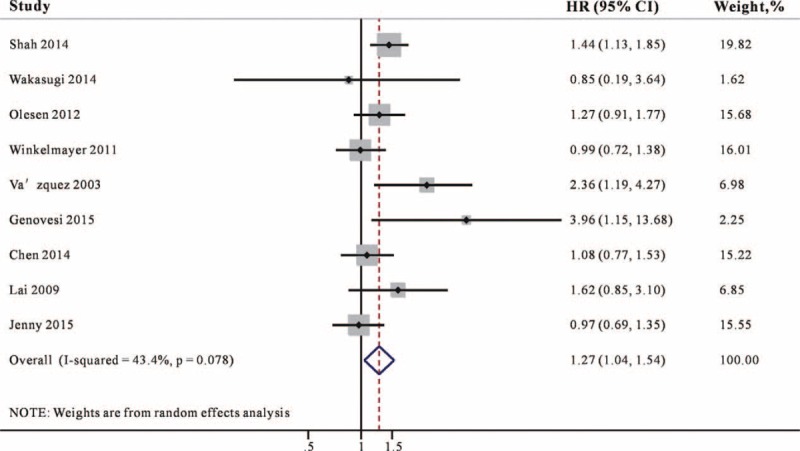
Combined estimate of hazard ratios (HRs) and 95% confidence intervals (CIs) of warfarin use associated with bleeding risk. Squares represent the HRs (size of square reflects the study's weight), and lines represent the 95% CIs for individual studies. The diamond represents the pooled HR and 95% CI. CI = confidence intervals, HRs =  hazard ratios.

Nine studies^11–17,20,21^ reported the association between warfarin and bleeding risk and the majority of studies controlled the potential confounders. In the meta-analysis of bleeding risk, a pooled analysis of 9 studies showed that warfarin use was associated with a 27% higher risk for bleeding (95% CI 1.04–1.54, Figure 3) compared with no-warfarin use. No heterogeneity was detected for this outcome (*I*^2^ = 43.4%).

### Association of Warfarin Use and All-Cause Mortality

**FIGURE 4:**
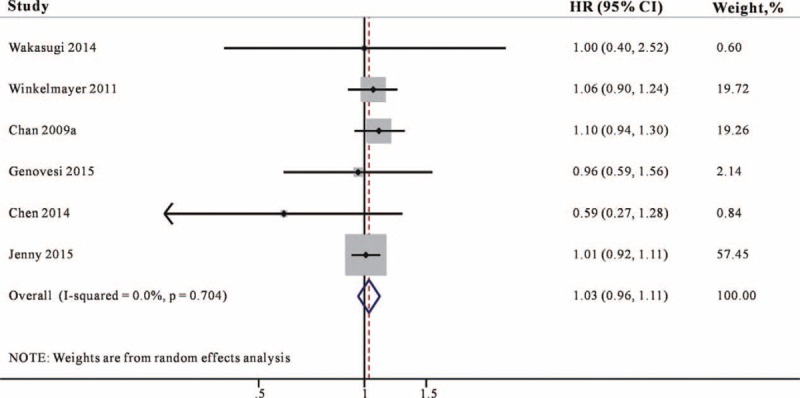
Combined estimate of hazard ratios (HRs) and 95% confidence intervals (CIs) of warfarin use associated with mortality risk. Squares represent the HRs (size of square reflects the study's weight), and lines represent the 95% CIs for individual studies. The diamond represents the pooled HR and 95% CI. CI = confidence intervals, HRs =  hazard ratios.

Six studies^11–13,15,17,19^ reported the association between warfarin use and the risk for mortality in AF patients undergoing dialysis. As demonstrated in Figure 4, warfarin use did not associate with statistically significant decreases in mortality (HR 1.03, 95% CI 0.96–1.11) and no significant heterogeneity was found (*I*^2^ = 0%, *P* = 0.704).

### Publication Bias

Funnel plots in the meta-analysis of stroke and bleeding appeared small asymmetric through visual examination, whereas Egger's tests indicated no significant publication bias (stroke, Egger's test: *P* = 0.815; bleeding, Egger's test: *P* = 0.518).

## DISCUSSION

In this meta-analysis of 11 observational studies, involving a total of 25,407 dialysis patients with AF, warfarin use did not reduce the risk for stroke and mortality but was associated with a 27% higher risk for bleeding. Thus, the risk–benefit ratio does not appear to be favorable to support a recommendation of routine warfarin use for stroke prevention in dialysis patients with AF. Dialysis patients with AF, compared with sinus rhythm, were associated with a greater risk for stroke and mortality.^5^ In routine practice, warfarin is widely used in dialysis patients with AF for stroke prevention though the ratio of risks to benefits remains unclear. In the current guideline for management of AF, the CHADS2 and CHADS2-VASc risk model scoring systems were dominantly used for anticoagulation decision-making to prevent stroke in AF; however, it does not make any specific mention of patients with ESRD.^25,26^ Thus, the risk–benefit ratio of warfarin in this clinical disorder should be assessed adequately. Recently, 2 published systematic reviews suggested that the majority of studies do not support a protective effect of warfarin in ESRD patients with AF.^27,28^ We assembled a synthesis of the available evidence on the efficacy and safety of warfarin treatment in dialysis patients with AF. The results of this study were consistent with a recently published meta-analysis that showed that warfarin use had no beneficial effect on reduction of stroke events but was associated with higher risk for bleeding.^29^ Dialysis patients have the intrinsic platelet dysfunction and altered platelet–vessel wall interaction secondary to uremia. ^30,31^ In addition, warfarin-induced vascular calcification through the inhibition of Matrix Gla protein and Gas-6 was also the important potential mechanism of the enhanced risk for ischemic stroke in dialysis patients.^32,33^ Furthermore, dialysis patients also experienced 4- to 10-fold higher rates of bleeding complication.^34^ Previous studies have showed that advanced age and comorbidities such as hypertension, heart failure, diabetes mellitus, and cerebrovascular disease were independently associated with increased risk of bleeding events during warfarin treatment,^35^ and are highly prevalent in the ESRD population.^36^ Indeed, these comorbidities were common among the patients included in this meta-analysis. In addition, dialysis patients routinely receive heparin during dialysis procedures, which also increases the risk for bleeding.^37^ These factors could explain why, in our study, warfarin was not associated with a lower risk for ischemic stroke, but was associated with a 27% increased risk for bleeding in dialysis patients with AF, which is higher than that reported in nondialysis patients (19%).^14^

Two studies included in the meta-analysis showed that that warfarin use was associated with a markedly decreased risk of stroke. In the study by Olesen et al,^16^ AF patients requiring dialysis were found to have a lower vascular events and mortality compared with non-ESRD, and the prevalence rate of coronary artery disease (27.5%), chronic heart failure (19%), hypertension (53.9%), and diabetes (14.3%) were relatively low among patients requiring dialysis. These mismatches may lead to selection bias; in that included “healthier” patients undergoing dialysis. In addition, the study conducted by Lai et al estimated an unadjusted HR and did not control the potential confounders, which may bias the results.^20^ These above factors could influence the true association between warfarin and stroke risk in dialysis patients with AF. We must emphasize here that all the data available on the subject are observational in nature. There are no randomized clinical trials that are specifically designed to evaluate the risk–benefit ratio of warfarin in dialysis patients with AF. This highlights the tremendous limitation in applying the current evidence to health care decisions. A decision to continue or start warfarin in a patient on dialysis should be considered carefully. This meta-analysis did not support that all AF patients undergoing dialysis are suitable for anticoagulation therapy based on current risk model scoring systems because the information on the distribution of AF types (paroxysmal, persistent, or permanent) in the patient populations were unavailable. A cross-sectional study reported that 67% of the documented AF in dialysis patients was paroxysmal.^38^ In addition, the decline in the renal function has found to be associated with the progression of AF from paroxysmal to persistent or permanent form.^39^ Therefore, it might be expected that patients with a greater number of episodes of AF and high-stroke risk would find greater benefit from warfarin therapy. Recently, an individualized risk stratification was recommended that included bleeding diathesis consideration, CHADS2 scoring system, antiplatelet therapy, and the risk factors for calciphylaxis, including obesity, systemic inflammation, diabetes mellitus, corticosteroid use, and protein C or S deficiency if warfarin is not used.^40^ It might provide a useful step toward informed decisions about warfarin use. Several limitations of this meta-analysis must be considered. First, several studies enrolled prevalent AF (existing diagnosis) but did not give detailed information about whether patients with prior warfarin use were excluded in the nonwarfarin group.^15,18,19^ As warfarin discontinuation often happens after a bleeding event or from perceived bleeding risk, such patients increase the risk of bleeding in the nonwarfarin group and diminish the risk associated with warfarin. Second, these included studies did not provide the data on the time in the therapeutic range for warfarin users and adequate data on the type of stroke. When dialysis patients cost a low amount of time in the therapeutic range or when cryptogenic and hemorrhagic strokes are more common in dialysis patients, the benefits will be diminished, which leads to a lack of efficacy of warfarin in this meta-analysis. Third, there is a significant heterogeneity in stroke risk across these studies. We consider that there are many unmeasured confounding, including different methods of dialysis, duration and frequency of maintenance dialysis, and heparin use which are all associated with thromboembolic risk and bleeding,^41^ may bias the results. Although the random-effects pooling method adjusts for heterogeneity, overall results can be fraught with significant heterogeneity and should thus be viewed with caution and as hypothesis generating.

## CONCLUSION

According to the currently existing evidence, warfarin use was not associated with a reduced risk of stroke but with a higher risk of bleeding in dialysis patients with AF. Adequately powered randomized trials are needed to determine the risk–benefit ratio of warfarin in these patients.
